# CD73: A Promising Biomarker in Cancer Patients

**DOI:** 10.3389/fphar.2020.609931

**Published:** 2020-11-16

**Authors:** Roberta Turiello, Aldo Pinto, Silvana Morello

**Affiliations:** Department of Pharmacy, University of Salerno, Fisciano, Italy

**Keywords:** CD73, adenosine, immunotherapy, cancer, biomarker

## Introduction

Over the past years, the adenosine pathway has become a topic of great interest in cancer research, due to an increasing number of evidences showing its role in tumor progression and metastases. Within tumor microenvironment, extracellular adenosine reaches elevated concentrations and by activating the adenosine receptor subtypes A2A and A2B limits the effector T cell functions, induces immunosuppression, and stimulates angiogenesis (recently reviewed in Allard et al., 2020). Extracellular adenosine production is finely regulated by many enzymes (Yegutkin, 2008), and it is critically impaired in pathological conditions, such as inflammatory disorders or cancer (Allard et al., 2020). The classical pathway of extracellular adenosine production is based on sequential reactions mediated by two ectonucleotidases, specifically extracellular adenosine triphosphate is first hydrolyzed by CD39 into adenosine monophosphate (AMP) and then dephosphorylated into adenosine by CD73. The alternative pathway involves CD38 and CD203a, which convert NAD^+^ into ADP-ribose and ADP-ribose into AMP, respectively (Horenstein et al., 2013). AMP is, in turn, dephosphorylated into adenosine by CD73. In this context, CD73 has aroused particular interest, being the key enzyme in extracellular adenosine production, both in classical and alternative pathways. CD73 is a dimeric enzyme that exists in two forms: one form is anchored, *via* glycosylphosphatidylinositol, to the membrane of many cells or extracellular vesicles; the second form is generated upon cleavage from membranes through the action of proteases or phospholipases, and it is found in biological fluids (Zimmermann et al., 2012).

A large number of preclinical studies have investigated the role of CD73 in immunosuppression and tumor progression, proving that the inhibition of CD73 is an effective immunotherapeutic strategy for different types of cancers. Agents targeting CD73, including monoclonal antibodies and small molecules, have been developed and are undergoing clinical trials, alone or in combination with other immune checkpoint inhibitors (Allard et al., 2020; Thompson and Powell, 2020). New CD73 inhibitors have proved to be effective in controlling tumor growth and immune response in preclinical studies and would warrant clinical investigations (Jin et al., 2020; Schäkel et al., 2020).

To date, various studies have also explored the significance of CD73 expression and activity in cancer patients, evaluating possible correlations with survival and/or clinical response. Here, we discuss the relevance of different forms of CD73 as prognostic biomarker of tumor progression in cancer patients or as predictive biomarker of response to anticancer therapies.

## Membrane-Bound CD73 in Cancer Patients

In cancer, CD73 is expressed by many subsets of cells populating the tumor lesion, including tumor cells, stromal cells, and endothelial cells, as well as infiltrating immune cells (Vijayan et al., 2017). High CD73 tumor expression is associated with shorter overall survival and poor prognosis of patients with melanoma (Monteiro et al., 2018), diffuse large B-cell lymphoma (Wang et al., 2019), breast cancer (Loi et al., 2013; Turcotte et al., 2017; Buisseret et al., 2018; Jiang et al., 2018), ovarian cancer (Turcotte et al., 2015; Jiang et al., 2018), head and neck cancer (Mandapathil et al., 2018), head and neck squamous carcinoma (Ren et al., 2016) non–small-cell lung cancer (Inoue et al., 2017), thyroid carcinoma (Bertoni et al., 2019), pancreatic cancer (Chen et al., 2020), gastric cancer (Lu et al., 2013), or colorectal cancer (Wu et al., 2012). Of note, the up-regulation of CD73 in cancer patients has been addressed as a mechanism of resistance to antitumor therapies. Loi and coworkers observed that CD73 gene expression significantly related to poor prognosis in triple-negative breast cancer patients treated with anthracycline-only preoperative chemotherapy (Loi et al., 2013). In the same study, in a breast cancer mouse model, the authors demonstrated that CD73 overexpression on tumor cells determines chemoresistance to anthracycline treatment, while the blockade of the enzyme improved antitumor immune response (Loi et al., 2013). In another study, CD73 expression associated with poor outcome of breast cancer patients treated with trastuzumab, an anti-HER2/ErbB2 antibody, while its expression on tumor cells and host cells was linked to resistance to monoclonal antibody treatment in a mouse model of HER2/ErbB2-driven breast cancer (Turcotte et al., 2017). Interestingly, a dynamic regulation of CD73 expression was hypothesized as an acquired mechanism of resistance to immunotherapy by Reinhardt and colleagues, who reported that melanoma patients who showed progressive disease during anti-PD-1 therapy had also increased CD73 expression in tumor tissue (Reinhardt et al., 2017). Patients who had prior therapy with inhibitors of MAPK and BRAF were negative for CD73 expression, while patients who had not received MAPK inhibitor therapy showed CD73 up-regulation at progression (Reinhardt et al., 2017). Although these results were obtained in a small number of patients and further investigations are required, they suggest that the tumor expression of CD73 may change on treatment.

Opposite results were obtained in bladder cancer patients, in whom low CD73 expression associated with better survival (Wettstein et al., 2015; Koivisto et al., 2018). Although results by Koivisto and collaborators were obtained from a single retrospective study, they are of great interest underlining the importance to analyze the CD73 expression in each cell types within the tumor microenvironment. CD73 negative epithelial cells significantly associated with poor survival both in patients with non–muscle-invasive bladder cancer and muscle-invasive bladder cancer, while CD73 expression in stromal fibroblasts or lymphocytes had no predictive power (Koivisto et al., 2018). As the authors suggest, these results may be due to the role of endothelial CD73 in controlling the permeability of the blood vessels and the extravasation of leukocytes. However, in prostate cancer, Leclerc and coworkers observed that high levels of CD73 in normal adjacent prostate epithelium were significantly associated with shorter biochemical recurrence–free survival, while high levels of CD73 in the tumor stroma were associated with longer biochemical recurrence–free survival (Leclerc et al., 2016). In endometrial carcinoma, CD73 is down-regulated in carcinoma cells of poorly differentiated and advanced-stage disease, compared with normal endometrium and low-grade tumors, highlighting the protective function of CD73-derived adenosine on epithelial integrity in normal endometrium (Bowser et al., 2016). Thus, the loss of CD73 on epithelial cells may promote the tumor progression. Additional evidence on the controversial role of CD73 were given by Wang and coworkers, who observed that the lower expression of CD73 in blood vessels of glioma patients than in normal brain may cause damage to the blood–brain barrier, thus creating advantageous conditions for tumor growth (Wang et al., 2016).

In a very recent work, it has been observed that CD73 is highly expressed on cancer-associated fibroblasts in human colorectal cancers, and high CAF frequency in cancer tissues correlates with elevated CD73 activity and poor prognosis (Yu et al., 2020). In patients with glioblastoma multiforme undergoing anti-PD-1 treatment, Goswami and colleagues individuated the presence of CD73^hi^ immunosuppressive myeloid cell subsets, which may cause less T-cell infiltration in tumor microenvironment (Goswami et al., 2020). In sarcoma and breast cancer, tumor-infiltrating NK cells express high level of CD73, and the frequency of CD73^+^ NK cells in the tumor microenvironment correlates with larger tumor size in patients with breast cancer (Neo et al., 2020).

CD73 is expressed not only on cell populations composing the tumor microenvironment but also on circulating immune cells. High baseline percentage of circulating CD8^+^PD-1^+^CD73^+^ lymphocytes correlate with worse survival in malignant melanoma patients treated with nivolumab (Capone et al., 2020).

## Soluble CD73 in Cancer Patients

Since a soluble form of human CD73 was identified (Thompson et al., 1987; Coade and Pearson, 1989), many studies were settled to understand the role of this form in inflammatory and tumor processes. In 1989, Lal and colleagues reported that CD73 expression in serum of head and neck cancer patients was higher than that in healthy subjects (Lal et al., 1989). Interestingly, the enzymatic activity increased with the advancement in the stage of cancer (Lal et al., 1989). Of note, Lehto and Sharom in 1998 observed that the soluble form of CD73 is enzymatically more active than the membrane-bound variant (Lehto and Sharom, 1998). Nowadays, a great number of publications state that soluble CD73 expression and activity are increased in several human cancers (Huang et al., 2015; Morello et al., 2017; De Lourdes Mora-García et al., 2019; Gardani et al., 2019). All these evidence constitute the rationale for new interesting studies aimed to clarify whether CD73 could be used as a soluble biomarker in cancer patients. At this regard, in 2017, we found that CD73 activity in serum of melanoma patients correlates with overall survival, progression-free survival, and clinical response to nivolumab treatment (Morello et al., 2017). Very recently, Messaoudi and collaborators observed that patients with colorectal cancer liver metastases who had high levels of soluble CD73 had also shorter survival (Messaoudi et al., 2020). In the same study, expression of tumor CD73 is a stronger biomarker than the soluble form.

Soluble CD73 in plasma of metastatic breast cancer patients tends to increase after radiotherapy treatment, while its blockade reduces the irradiated tumor volume and, if combined with CTLA-4 blockade, inhibits lung metastases in a mice model (Wennerberg et al., 2020).

## CD73 on Extracellular Vesicles

The circulating portion of non–cell-bound CD73 also includes extracellular vesicles, and in particular, exosomes (30–150 nm) that can be produced by almost all cells, including cancer cells (Becker et al., 2016; Théry et al., 2018). The published studies focused on soluble CD73 in human fluids do not consider these vesicles, so it is still unclear whether the soluble CD73 expression and/or activity is influenced by the exosomal form. To date, no studies considering both the shedded and the exosomal forms have been published, and this represents a very interesting point to investigate on.

CD73 has been detected on human exosomes isolated from plasma and serum (Muller et al., 2014; Schuler et al., 2014; Theodoraki et al., 2018) and pleural fluid (Clayton et al., 2011). Notably, CD73 expressed on exosomes maintains its enzymatic activity, and the exosome-derived adenosine is responsible of T-cell inhibition and impaired antitumor immune response (Clayton et al., 2011; Schuler et al., 2014; Ludwig et al., 2017; Zhang et al., 2019). Expression of CD73 and CD39 on exosomes isolated from plasma of HNSCC patients related with the stage of disease, being higher in stage III/IV than stage I/II (Theodoraki et al., 2018).

Exosomes isolated from UMSCC47 cell lines are not only able to produce adenosine, *via* CD39 and CD73, but also carry adenosine and inosine in their inner compartment (Ludwig et al., 2020). Thus, exosomes can circulate in body fluids and can promote a tumorigenic environment by producing adenosine in loco, *via* CD39 and CD73, and also by transporting and releasing adenosine and inosine far from the site where they are released, protecting these molecules from metabolism or uptake processes (Ludwig et al., 2020). This could be one of the mechanisms by which exosomes promote tumor growth and metastases dissemination.

## Conclusion and Future Perspectives

The purinergic signaling plays a critical role in cancer, since adenosine triphosphate is released in the tumor microenvironment upon hypoxia, nutrient starvation, cell death, or treatment with some chemotherapeutic agents. It is well known that adenosine triphosphate acts as pro-inflammatory mediator; nevertheless, the high expression of CD39 and CD73 in tumor microenvironment enhances its conversion into adenosine, which, in turn, is responsible for tumor growth and impaired immune response (Sorrentino et al., 2013).

Many components of the adenosine pathway have been assessed as a therapeutic target or potential biomarker of prognosis. In particular, CD73 has emerged as a promising candidate both as a target and biomarker in different human tumors, as mentioned above, helpful in clinical practice to select patients that would likely respond to CD73-targeted therapy. Many studies focusing on the expression of CD73 in tumor tissue have been published; nevertheless, it has to be noted that results may be different according to the tissue type and its heterogeneity, or to the technics used to measure the expression. Furthermore, the possible effect of pharmacological treatments on CD73 expression needs to be considered. The analysis on tumor tissue is an invasive and painful procedure that could be particularly difficult in patients with metastatic disease. The analysis of the soluble CD73 in biological fluids, which needs further investigations, could represent an additional tool in clinical practice.

To date, further investigations are required to better understand the role of CD73^+^ exosomes in cancer, evaluating the possibility to use these vesicles as biomarkers of prognosis. One more point that needs to be explored is the mechanism regulating the cleavage of CD73 from the cell membrane. It could be of great interest to fully understand the stimuli that promote the shedding and/or the production of CD73^+^ exosomes, evaluating whether the blockade of these processes could impact tumor progression.

## Author Contributions

RT and SM conceived the manuscript. RT, AP, and SM drafted and reviewed the manuscript.

## Conflict of Interest

The authors declare that the research was conducted in the absence of any commercial or financial relationships that could be construed as a potential conflict of interest.
